# Supplementary figures and images for: PKA regulatory subunit Bcy1 couples growth, lipid metabolism, and fermentation during anaerobic xylose growth in Saccharomyces cerevisiae
Source: PLoS Genet. 2023 Jul 6;19(7):e1010593. doi: 10.1371/journal.pgen.1010593 (PMC10353814; doi:10.1371/journal.pgen.1010593)

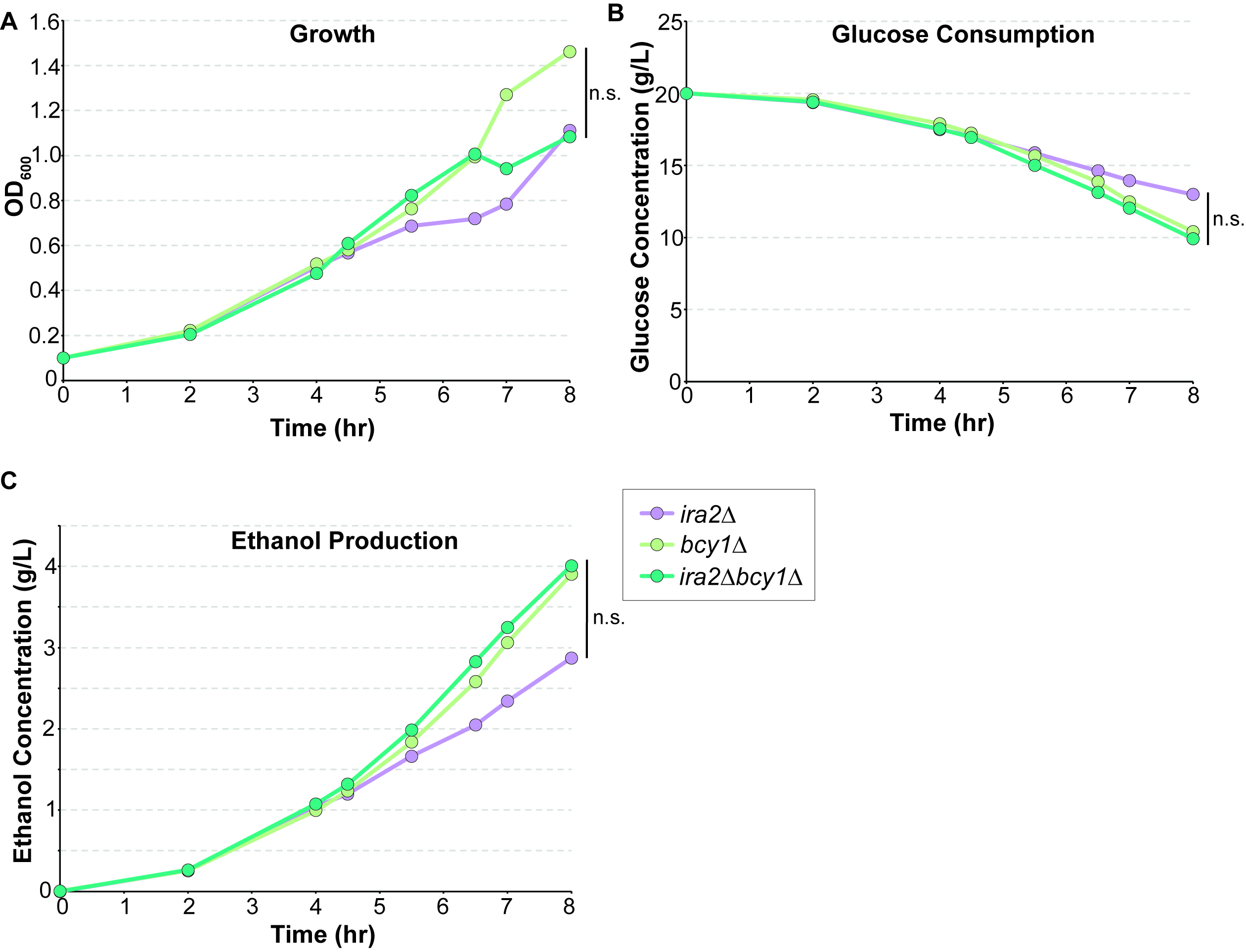

Supplement: S1 Fig — A-C. Average (n = 3 biological replicates) (A) growth (OD600, optical density), (B) glucose concentration, and (C) ethanol concentration of ira2Δ, bcy1Δ, and ira2Δbcy1Δ strains grown anaerobically on rich glucose medium (p > 0.05, ANOVA). (TIF) [file pgen.1010593.s001.tif]

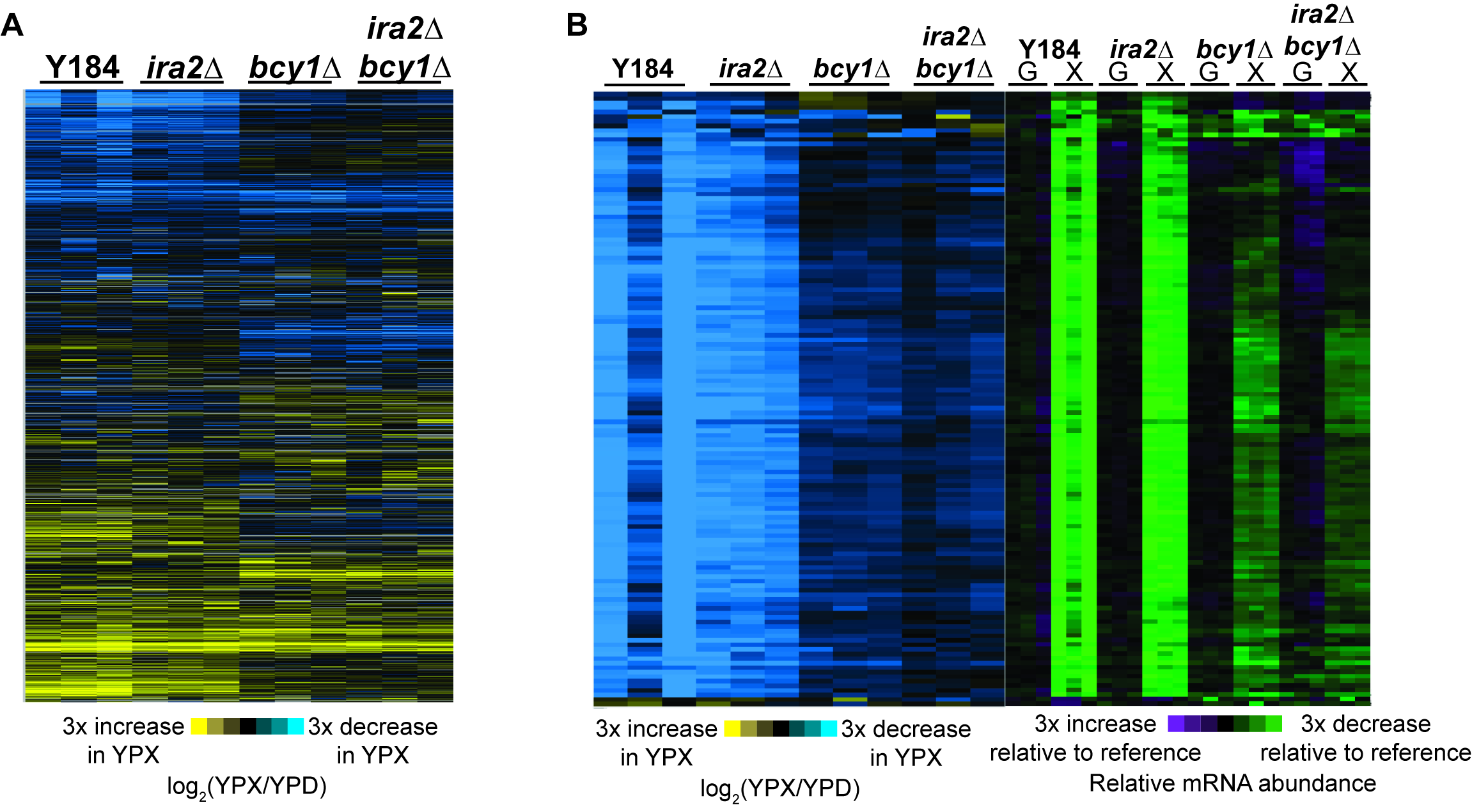

Supplement: S2 Fig — A. Expression of 5834 genes (rows) detected in all four strains (Y184, ira2Δ, bcy1Δ, ira2Δbcy1Δ), organized by hierarchical clustering of log2(fold change) upon glucose-to-xylose shift, as described in Fig 2. Each column represents one of three biological replicates of the denoted strain listed above. B. Expression of 135 ribosomal protein genes (rows) in all four strains (Y184, ira2Δ, bcy1Δ, ira2Δbcy1Δ), organized by hierarchical clustering of log2(fold change) upon glucose-to-xylose shift. The blue-yellow heatmap on the left represents the log2(fold change) in expression upon glucose to xylose shift across biological triplicates (columns). The purple-green heatmap on the right represents the abundance of each transcript (rows) in each strain grown on glucose (G) or xylose (X), relative to the average (n = 3) abundance of transcripts measured in the Y184 YPD sample. (TIF) [file pgen.1010593.s002.tif]

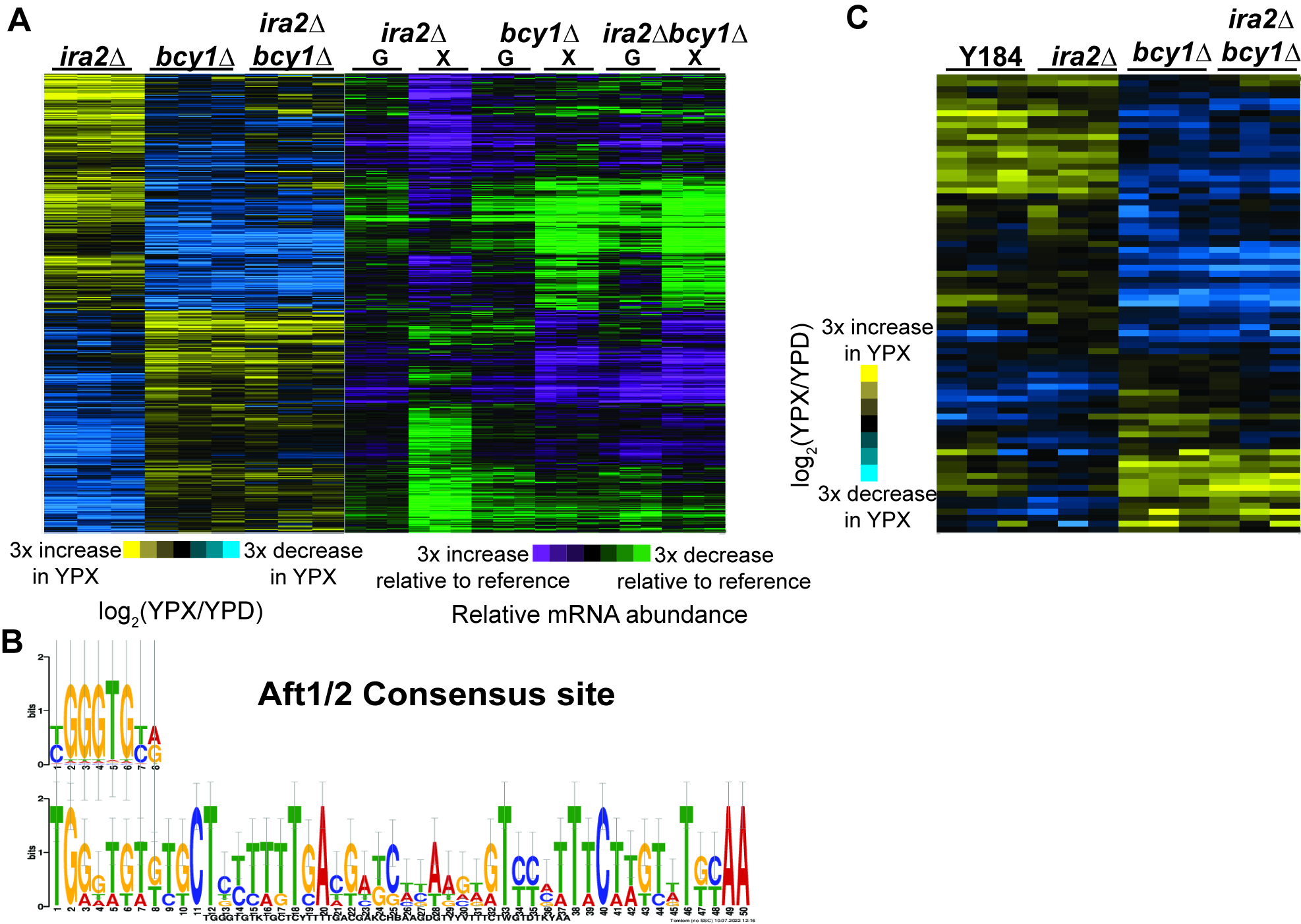

Supplement: S3 Fig — A. Expression of 654 genes whose log2(fold change) upon glucose to xylose shift is different (FDR < 0.05) between the ira2Δ and bcy1Δ strains and whose change in expression is in the opposite direction (increased or decreased) across strains. (see Methods for details). The yellow-blue heatmap on the left represents the YPX/YPD log2(fold change). The green-purple heatmap on the right represents transcript (rows) abundance in anaerobic glucose (G) and anaerobic xylose (X) relative to the average (n = 3) abundance of transcript in the Y184 YPD sample. B. ~500 base pairs upstream of the ORF for genes repressed in the bcy1Δ strain upon shift to xylose were analyzed for enriched motifs (bottom motif; MEME Suite), analyzed for known transcription factor binding sites (TOMTOM), and identified the Aft1/2 consensus site (top motif; see Methods for details). C. Expression of 77 genes from A whose promoters are physically bound by Ino2 and/or Ino4, organized by hierarchical clustering. (TIF) [file pgen.1010593.s003.tif]

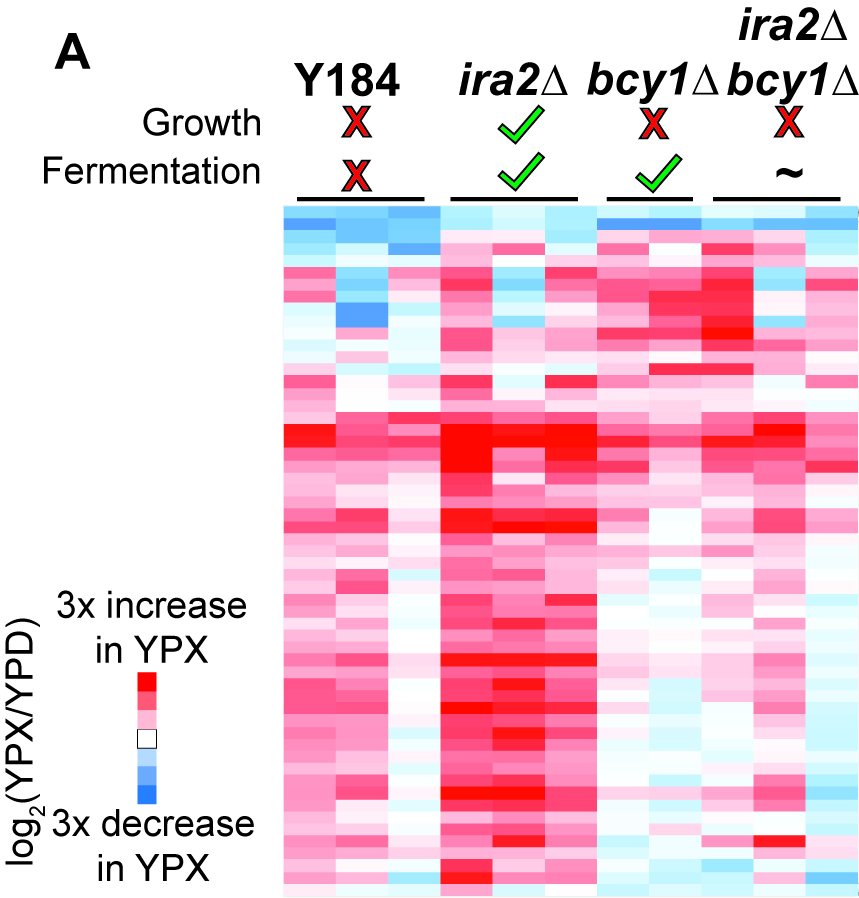

Supplement: S4 Fig — A. The majority of phosphatidylcholine species (rows) identified show significantly lower log2(fold change) upon the shift from glucose to xylose in the bcy1Δ and ira2Δbcy1Δ compared to the ira2Δ strain (p = 0.0015082, ANOVA). (TIF) [file pgen.1010593.s004.tif]

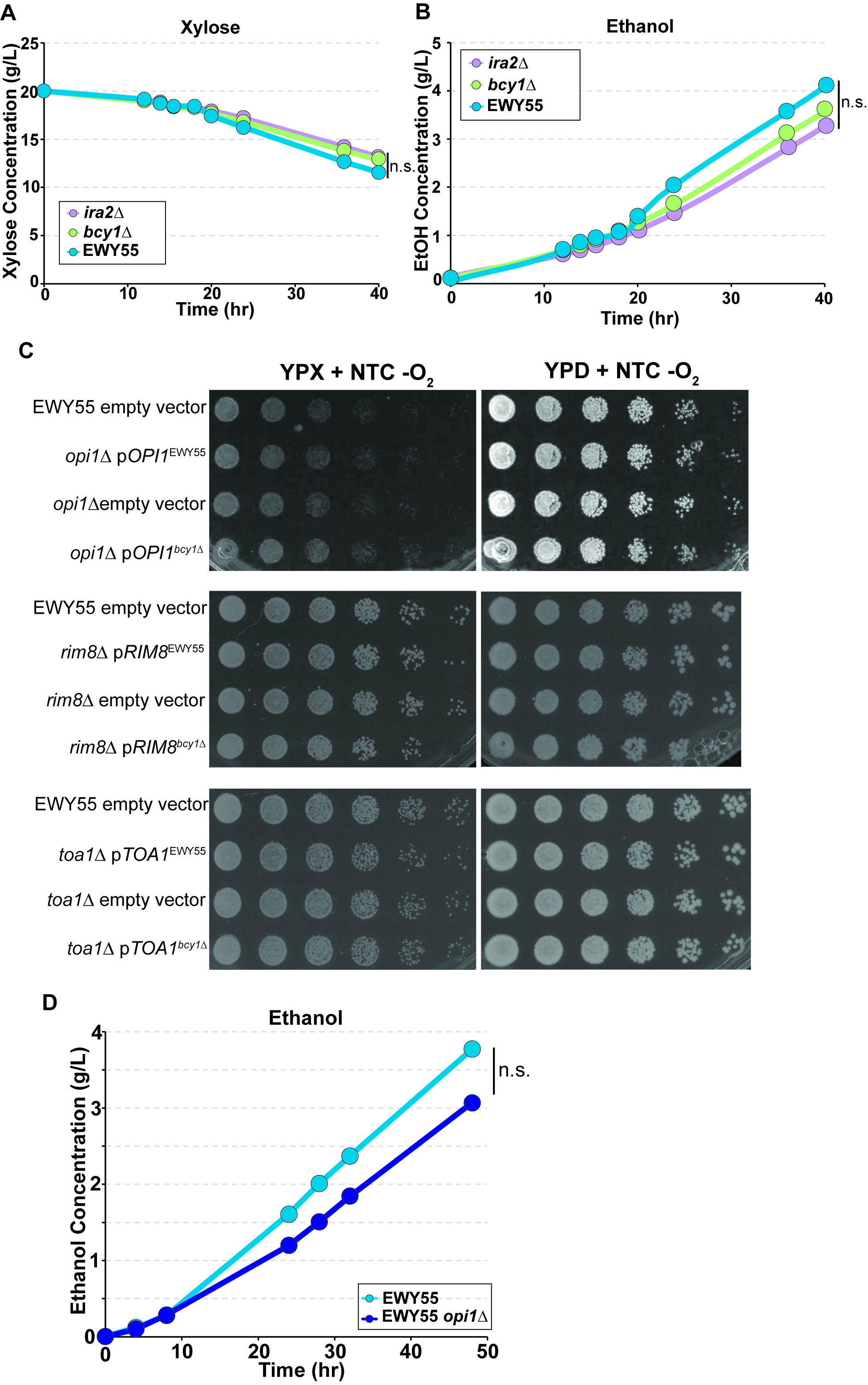

Supplement: S5 Fig — A-B. Average (n = 3 biological replicates) of (A) xylose concentration or (B) ethanol concentration for ira2Δ, bcy1Δ, and EWY55 strains anaerobically grown in rich xylose medium (p > 0.05, ANOVA). C. Representatives of multiple replicates of EWY55 or EWY55 cells lacking OPI1 (top panels), RIM8 (middle panels), or TOA1 (bottom panels) and complemented with an empty vector or parental or evolved allele grown anaerobically on solid xylose (left) or glucose (right) medium with NTC selection. D. Average (n = 3 biological replicates) of ethanol concentration for EWY55 and EWY55 opi1Δ strains anaerobically grown in rich xylose medium (p > 0.05). (TIF) [file pgen.1010593.s005.tif]

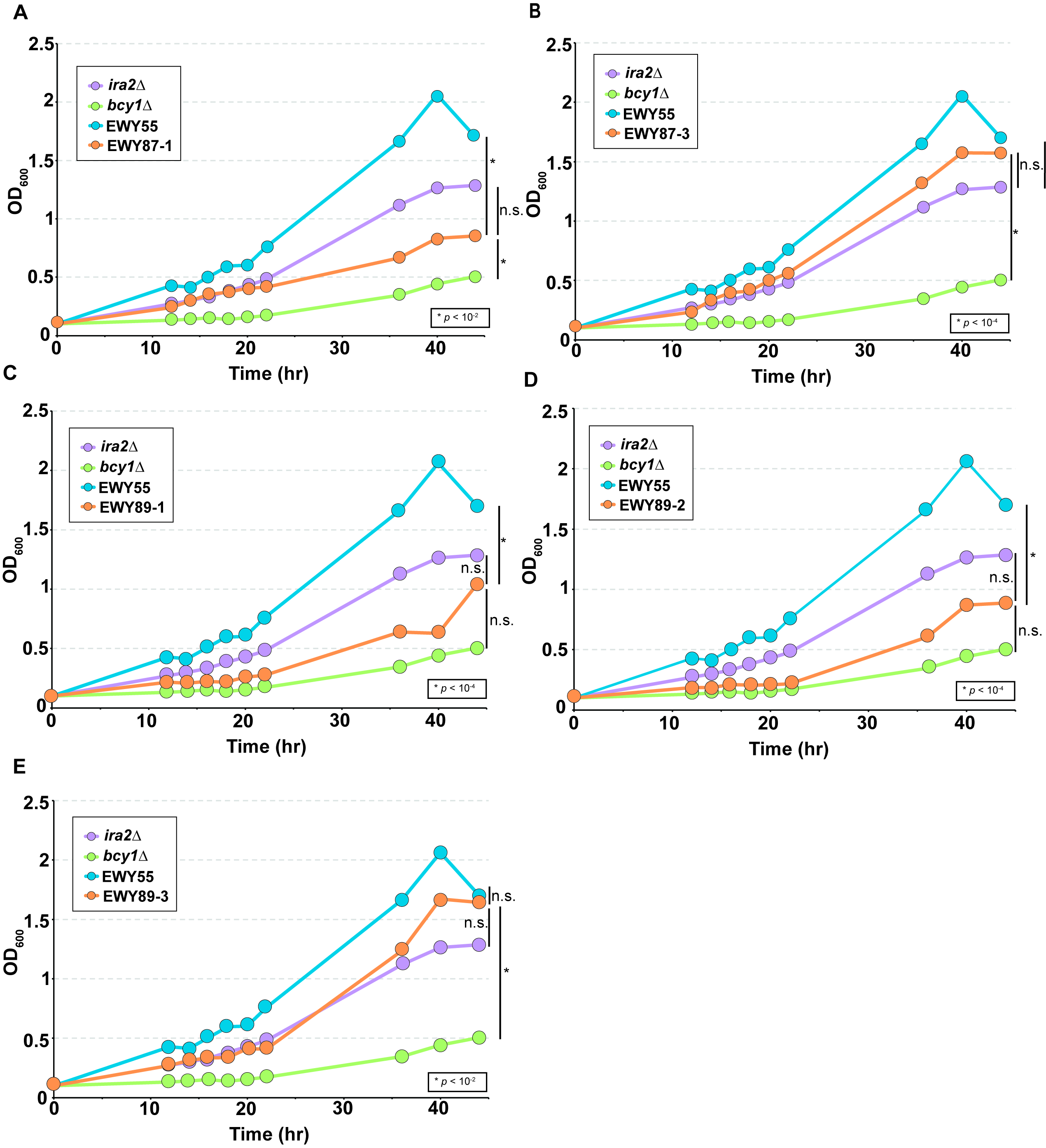

Supplement: S6 Fig — A-E. Average (n = 3 biological replicates) growth (OD600, optical density) of ira2Δ, bcy1Δ, EWY55, and (A) EWY87-1, (B) EWY87-3, (C) EWY89-1, (D) EWY89-2, or (E) EWY89-3 strains grown anaerobically on rich xylose medium (p < 10−4, ANOVA). (TIF) [file pgen.1010593.s006.tif]
