# Supplementary material for: PKA regulatory subunit Bcy1 couples growth, lipid metabolism, and fermentation during anaerobic xylose growth in Saccharomyces cerevisiae
Source: PLoS Genet. 2023 Jul 6;19(7):e1010593. doi: 10.1371/journal.pgen.1010593 (PMC10353814; doi:10.1371/journal.pgen.1010593)
Supplement: Attachment — Submitted filename: Wagner_PlosGen-Bcy1_ResponseLetter.docx [file pgen.1010593.s024.docx]

Comment from the editor:
The reviews were positive, but did bring up many questions, clarifications and suggestions. While I found no key concern that must be addressed for publication, the reviewers comments are quite thoughtful and they have suggested both experiments and changes to the manuscript that would improve the quality and impact of the work.

Thank you very much for the positive assessment. As outlined below, we have addressed all of the reviewers’ comments, incorporating suggestions into our revisions and in some cases clarifying points of confusion. This includes several new experiments (Fig 1B-C, Fig S1, Fig 5) and new analysis or data presentations. These changes have strengthened the manuscript and we hope that you agree.

Reviewer's Responses to Questions

**Comments to the Authors:**

Reviewer #1: Signaling pathways control a diversity of cellular responses, from changes in cell morphology that occur during differentiation, to changes in cellular metabolism, to the response to stress. One terrific model to understand cellular signaling is the budding yeast S. cerevisiae because of the ease of genetic manipulation and the long history of characterization of its main signaling pathways. One of the major nutrient-regulatory pathways in yeast is the Ras-cAMP-PKA pathway. This pathway controls the cellular response to nutrients, particular carbon and nitrogen sources, by executing a wide variety of effector responses. In the study by Wagner et al., the role of the Ras-cAMP-PKA pathway is explored from the perspective of xylose production, a compound used in biofuels. An assortment of ‘omics’ approaches and directed evolution experiments identify proteins that couple xylose-dependent growth to metabolism.

The abstract, introduction, and discussion of the manuscript are for the most part well written and suitable for a general audience. Previous literature is cited and put into the context of the current study. In the results, multiple ‘genome-wide’ experiments were performed – transcriptomics, lipidomics, and proteomics. It is clear that these experiments appear to be carefully executed, and new biologically relevant connections have been uncovered by these approaches. For example, PKA appears to control a response to iron (known), carbon (known), fatty acid (new), and phospholipid metabolism (new). The main problem is that the manuscript comes across as more of a survey, because few of the new leads were followed up on. If one molecular mechanism were pinpointed or verified among the many suggested possibilities, it would strengthen the ms and would certainly be on par with standards of PLOS Genetics. For example, in Figures 2 and 3, the authors identify many potential routes for new regulation. Instead of pursuing these hypotheses, the authors do two more global experiments. Moreover, the two main hypotheses tested by specific experiments produced results that were ambiguous. One test in Figure 4B showed the effect of inositol on growth, which very modestly stimulated growth of the bcy1 mutant, and also appeared to stimulate growth of the ira2 mutant. The other test showed that loss of opi1 impacted the growth of an evolved strain, which for technical reasons could not be recapulated. The second major problem with the paper is the presentation of the data repeatedly forces the reader into the supplement. Reorganization of the figures may address this problem. Specific concerns are noted below.

We thank the reviewer for their positive assessments about the manuscript’s rigor, presentation, and novel results. We disagree with the reviewer that we did not follow up on main conclusions: we incorporated a multi-omics and systems biology perspective to present several major new insights, including those highlighted above by this reviewer. We agree that some details previously on the supplement are better represented in the main text and have moved several figures to the main text, as outlined below. Beyond this, our multi-omic perspective has pointed to several important results that highlight the role of cellular physiology (rather than individual genes); our newly added data regarding Opi1 also strengthens those conclusions, as outlined below. Overall, we agree with this reviewer about the important novel points of the work, and we believe that it will make a great contribution to *PLoS Genetics*.

Major points

Figure 1, the ira2 bcy1 double mutant is referred to in panel D but not panels B and C (it’s in the supplement). Perhaps this data can be shown in the figure to reduce confusion? Moreover, Fig. S1 shows ira2 and bcy1 single mutants show reduced xylose but an ira1 bcy1 double does not. How are the authors concluding the double mutant is fermenting xylose?

First, we apologize that the wrong figure was shown on the supplement; second, we agree with the reviewer that this result should be in the main body. We have now added the result to the main Fig 1B-C. These data were recollected as part of our revisions: the new data show that the *ira2bcy1* double mutant has a variable xylose metabolism phenotype (on some days consuming xylose anaerobically as the *bcy1-* strain and on other days not); but there is an invariant growth arrest under these conditions. As explained in the manuscript, it is possible that extremely high PKA activity can actually inhibit metabolism (as known for some other sugars); nonetheless, since the manuscript focuses on decoupling in the *bcy1-* strain, and since the *ira2bcy1* double mutant is mostly used to understand the genetics of PKA pathways, this result does not affected any of our conclusions. To be safe, we redid one analysis focusing on xylose metabolism (lines 238-256) – none of the original conclusions or results, in this section or elsewhere in the paper, are impacted by this.

Figure 2 is entitled ‘Few transcriptomic changes’ however, for the non-expert, it looks like many gene levels change. Our expectation might be that 65 genes and 603 genes is a lot. Where are the two genes that changed on the heat map (DSE4 and MET2)? Similarly, where is *CLN2* and *CDC20* on the heat map? For 63 genes, perhaps they all can be put alongside the heat map?

We change the title of this figure to “Few transcriptomic patterns correlate with anaerobic xylose growth” to clarify our point, which is that there are few expression changes whose expression patterns parallel the growth differences. For example, although there were 65 genes differentially expressed between the *ira2-* strain that can grow on xylose and the other strains that cannot, the directionality of their expression changes in the non-growing *bcy1-* and *ira2bcy1* strains clearly do correlate with the growth phenotypes across the four strains. Thus the statement refers to the pattern of the genes’ expression and not the number of genes identified before directionality was taken into consideration. Likewise, the expression patterns of genes identified in the xylose fermenting strains versus Y184 do not correlate strictly with their fermentation patterns: once again, expression in the *ira2-* strain is most similar to Y184. We updated the text in several places to clarify our rationale and conclusions.

RAS pathway comments:
a. Lines 617-619: “One possibility is that *BCY1* deletion upregulates PKA activity to a higher level than deletion of *IRA2*, whose activation of PKA is indirect via cAMP regulation.” PKA is also regulated by the GPCR-G-alpha protein branch (encoded by GPR1 and GPA2). Perhaps the authors could also comment on this playing a role, or why an *ira2* deletion is not equal to a *bcy1* deletion.

We thank the reviewer for this useful point. We included a short section in the Discussion about why the *IRA2* and *BCY1* deletions may have different phenotypes, including their function in different branches of the pathway (lines 667-673).

b. The Ras pathway and the TOR pathway both control ribosomal function in response to nutrient stress and maybe should be mentioned in the paper and cited: Kunkel, J., Luo, X. & Capaldi, A.P. Integrated TORC1 and PKA signaling control the temporal activation of glucose-induced gene expression in yeast. Nat Commun 10, 3558 (2019). <https://doi.org/10.1038/s41467-019-11540-y>

c. This paper might also be of interest to the authors, as a similar compendium of data was generated surrounding the same pathway, and the authors (somehow) managed to tie the ‘big data’ to real biology together: Multi-omics analysis of glucose-mediated signaling by a moonlighting Gβ protein Asc1/RACK1

We thank the reviewer for these suggestions. We added citations to these papers in the Discussion (lines 667-673).

The authors refer to “(Fig 2C, Cluster II)” Maybe add the following “(Fig 2C, Cluster II, see II at right of figure), for clarity. Again, for Fig 2C, the authors refer to genes not shown in the figure, forcing the reader to go to the supplement to understand the figure.

The updated figure highlights specific clusters discussed in the text and annotates specific genes. In addition, in response to this and other comments of this reviewer we added a new Figure 2D that shows metabolism gene expression patterns directly.

Are any of the phosphor-proteomic data biologically relevant?

This is an important question and a difficult one to answer without other data. For important phosphorylation events, we highlight in the main text when their functions are known. In some cases, functions are not known but show genetic patterns of interest (*e.g.* dependence on PKA signaling).

a. The statement “PKA has been implicated in Azf1 phosphorylation (60)”. Does PKA phosphorylate Azf1? What was the actual finding uncovered by this global study?

Our past work showed that over-expressing *AZF1* in the *ira2-* strain improves anaerobic xylose fermentation. Here, we find that Azf1 targets are naturally higher expressed in the *bcy1-* strain.

That our analysis identified a known regulator of the phenotype gives credence to our work, bolstering other links to novel regulators made in this analysis. The study cited here found in a high-throughput experiment that Azf1 was phosphorylated by PKA, although the precise function of the phosphorylation event is not clear.

b. “Together, these results indicate PKA-dependent inhibition of PS synthesis in Y184 cells.” Does the phosphorylation mean anything? Does it occur in a regulated manner?

c. Does phosphorylation on S823 on Pah1 serve a function seeing as it has not been previously noted as a phosphorylation site?

These are interesting questions that will require future work to dissect. Our point in highlighting these responses is that we have identified previously uncharacterized phosphorylation events on proteins implicated by our ‘omic analysis, elevating their prioritization for future studies.

The authors often refer to ‘integrated signaling systems’; however, another possibility is that there is a cascade of failures that leads to multiple problems that causes the induction of one gene set and then another. This may not be ‘integrated’ even though it is global.

We appreciate this possibility raised by the reviewer. The reason we refer to ‘integrated’ signaling systems is influenced by our past work showing that rewiring of multiple pathways known to intersect is required for anaerobic xylose fermentation.

In Figure 4A, the genes could be labelled to help the reader see what lipids are changing.

We considered this in making the figure, but it is too challenging to read; therefore, we highlight the main lipids discussed in the paper and provide the full dataset for the figure on the supplement for interested readers.

Figure 4C is necessary to show the whole pathway but needs work to make it clear to the reader.
a. Why are some blue and yellow squares sharp and other hazy?
b. What does the term CL in the pink square mean?

We apologize for the confusion. We updated the figure legend and parts of the figure to clarify. The updated figure legend explains that sharp versus hazy boxes correspond to statistical significance and CL refers to cardiolipin. We hope that the figure is now clearer.

There is a problem with the statement “*bcy1Δ* cells experienced a very modest but statistically significant growth improvement (p = 2.4 x 10-6, ANOVA; Fig 4D), whereas the *ira2Δ* strain did not.” In the figure the *bcy1* mutant goes from 0.59 to 0.8 (0.21 difference). The *ira2* mutant similarly goes from 1.3 to 1.42 (0.12). This difference was visually obvious although not statistically significant and should be at least mentioned in the text.

Respectfully, the *ira2-* effect is not only not statistically significant but the effect is in the opposite direction as the *bcy1-* strain. Thus, our claims in the text are born out that the *bcy1-* strain experiences a very modest growth improvement whereas the *ira2-* strain experiences worse growth, although not statistically significant.

Lines 427-430: “While the bcy1∆ strain’s inability to grow anaerobically on xylose cannot be fully explained by a deficiency in phospholipid precursors, the modest improvement implicates it as a contributing factor to the phenotype. Why is the effect of supplementing inositol and choline so late in the time course? If this was to explain partially why the bcy1∆ strain grows more poorly than the ira2∆ strain in xylose, then shouldn’t supplementation allow the strains to grow similarly in the early part of the time course and remain similar for longer than without supplementation?

We are not sure why the very modest effect is most detectible midway through the time course; our main point in this figure is that supplementation does not provide a major correction to the growth rate.

The directed evolution experiments are interesting but the interpretations can be tricky.
a. For example, the change that was identified in Opi1 resulted in an amino acid substitution (S239A), yet the comparison explored was a whole-gene deletion of Opi1. Given the subtlety of the growth defect, might it be better to plot the growth of strains instead of dilutions shown in figure 5B, as has been done for other strains in the paper?

We redid this work in liquid cultures and now present data that is much more conclusive than previously shown. We replaced the previously shown spot assay with the results from liquid cultures that show growth rate, xylose concentration, and ethanol concentration over time (new Fig 5B and S5 Fig D). It is now clear that the EWY55 *opi1∆* strain has statistically significantly reduced anaerobic growth on xylose compared to the EWY55 strain but retains robust anaerobic xylose consumption and ethanol production – thus deletion of *OPI1* from the evolved strain partially reverts the phenotype to the original *bcy1-* parent. These results are much clearer than shown previously and significantly bolster our conclusions that Opi1 and phospholipid metabolism are involved in the decoupling of growth and metabolism in the *bcy1-* strain.

b. The authors concede this point and refer to strain manipulation problems. Lines 488-490: “Complementation experiments were not successful, since introducing even the empty vector into this strain complemented anaerobic xylose growth on a plate for reasons that are not clear but may suggest that the cells grow differently during drug selection.” Have the authors tested an auxotrophic marker instead of an antibiotic marked plasmid to verify this hypothesis?

We thank the reviewer for this suggestion. We worked for months on many avenues to get this assay to work and unfortunately have exhausted options at this point. Given that the genetics of the trait is likely to be complex, we have opted to address these limitations in the text. We highlight that the new EWY55 *opi1∆* results above significantly implicate Opi1 function in the trait.

c. Lines 483-484: “This strain background is derived from a wild isolate that is less genetically amendable than laboratory strains” Do the authors have any other strain backgrounds they can try and verify this in?

Due to the number of genetic modifications used in this strain engineered for non-native sugar fermentation, recapitulating the xylose fermenting background is unfortunately beyond the scope of this publication at this point.

d. Line 486: “nor did substituting the parental alleles into the evolved strain” Have the authors tried the opposite way by substituting the mutated alleles into the parental bcy1∆ strain? This seems like a better approach to test each mutation one at a time because EWY55 has multiple mutations and a chromosomal duplication.

We did try this as one of our many approaches and were unable to detect a phenotype, as described in the text.

e. The fact that aneuploidy occurs in the evolution experiments more often than not may suggest CNV could play a role instead of a SNP mutation. Have the authors looked at what genes are present on the duplicated chromosomes to see if they may help explain the phenotype? Also, have the authors ruled out whether aneuploidy affects growth in general?

This is an interesting question and one that we briefly looked into; however, given the number of genes on the amplified chromosome and the fact that aneuploidy frequently occurs stochastically in wild strains, we cannot speculate if there are specific genes that could be influencing the phenotype.

f. For Fig 5B, does deleting *OPI1* also decrease growth if done in the parental strains? It could be that deleting *OPI1* in any strain may reduce growth and may not be specific to EWY55. If this is the case, then deleting *OPI1* in EWY55 alone is not sufficient to suggest that the allele is a causal gene for the phenotype. In that case, I think the authors should generate the *OPI1* allele by making the SNP change in the parental *bcy1∆* strain using CRISPR-Cas9 to verify that it is playing the role they suggest it is.

Our new experiment addresses this point in a different way: we now include not only growth profiles but also xylose fermentation profiles in the EWY55 strain lacking *OPI1*. Our new results show that deletion of the evolved *opi1* allele from EWY55 reduces anaerobic growth on xylose – but it does not affect the robust anaerobic xylose fermentation. Thus, the gene deletion partially reverts the phenotype to that of the *bcy1-* parent (which is already atypical and thus hard to test in a different strain). We clearly state in the text that we were unable to test if it is this specific allele of opi1 that contributes; but these new results with all the other implications in the manuscript of Opi1 and phospholipid function strongly suggest this pathway is involved.

g. Figure 5C is difficult to understand. To what other figure do clusters A, B, C, D refer to?

We apologize for the confusion, we changed Fig 5C to a heatmap instead of the boxplots and have removed two of the clusters, so that only clusters referred to in the text are highlighted in the figure.

Typos and Minor Comments

1. Figure 1, the term ‘Y184’ is not clear until the reader determines that this refers to the wild-type strain. Perhaps call it wild type and refer to the specific name in the legends.

We prefer not to refer to Y184 as “wild type” since it is heavily engineered. Instead, we refer to it as the “parental” starting strain for the evolution experiments to which other strains are compared.

2. Fig 2B is not referenced before 2C.

This has been fixed in the text.

3. “genetically amendable than laboratory strains” Usually the word used is amenable, although formally speaking this word choice is correct.

We changed the text to say “amenable”.

4. Typos in author summary: “These individual processes have been well study, but the coordination and crosstalk between the process is not well understood.” And “implicated altered regulatory mechanisms involved in lipid metabolism correlating with decouple growth and metabolism.”

These have been fixed in the text.

5. The term “shows expression changes in the opposite direction” seems clunky.

We altered the text in several places to better explain transcriptional changes that are in the opposite direction (*e.g.* induced instead of repressed).

6. The term ‘strain-specific differences’ implies that different strain backgrounds are being evaluated. However, what seems to be examined is wild type compared to mutant.

We thank the reviewer for pointing this out. We have changed the text to “mutant-specific differences.”

Reviewer #2: Wanger et al. performed a descriptive multiomics study deeply delving into the role of Bcy1, the PKA regulatory subunit, in yeast physiology on xylose in anaerobic culture conditions. BCY1 deletion strain cannot grow on xylose but metabolize it anaerobically due to defects in lipid homeostasis. Together with laboratory evolution, the authors additionally found that mutations in TPK1 encoding a PKA catalytic subunit and phospholipid biosynthesis regulator OPI1 recoupled the growth on and utilization of xylose of the bcy1 deletion strain, suggesting the important role of PKA-dependent regulation of lipid metabolism in the growth of the yeast strain on xylose in anaerobic conditions.

Major concerns

1.1. L145: For the comparisons between glucose and xylose cultures of these strains, the authors sampled 3 hours after the carbon source transition (glc to xyl). Did all the 4 strains exhibit similar growth/metabolites patterns on glucose before the transfer? Readers may wonder whether the differences in growth/metabolism/transcription patterns were mainly caused by the “xylose-responsive changes” or the pre-culture (on glucose) effect. For instance, one strain exhibited a significantly slower growth and/or glucose metabolism, and consequently, the strain was transferred to xyl-anaerobic culture during glucose utilization while other strains were transferred during ethanol utilization.

We thank the reviewer for this important point. We specified in the text and show in Fig 2B that there are few expression differences across strains when grown anaerobically on glucose. Furthermore, we now add growth curves of the strains growing in glucose in S1 Fig, and point the reader to previously studied glucose consumption and ethanol production rates in these strains, found in Wagner et al. *PLoS One* 2019. These results show that the vast majority of gene expression differences reflect differential expression during the anaerobic xylose phase. Furthermore, substantial differences in strain growth or metabolism before the xylose shift cannot explain the major differences in growth and metabolism after shift to xylose.

1.2. It would be great if the authors presented the glucose culture profiles tracking glucose consumption, ethanol production, and other metabolites, as well as the transition time point for the preculture experiment.

We have added plots to S1 Fig tracking growth, glucose concentration, and ethanol concentration over an 8 hour time course under anaerobic glucose conditions. Based on statistical analysis, there is no significant difference between any strains in growth, glucose consumption, or ethanol consumption.

2.1. Also, 3 hours after the carbon source transition (probably aerobic to anaerobic culture condition transition as well, according to L653) is long enough for the ira2 deletion strain to resume its growth on xylose but might not for other strains. This can lead to some confusion about the data and authors’ arguments in the manuscript (for instance, L182 and L218).

In our past work, we have done many long-term experiments with the *bcy1-* mutant and it does not grow even after long periods of anaerobic xylose growth. Thus, our conclusion that the strain shows limited growth under these conditions is validated over long time frames. A statement addressing this has been added to the first paragraph of the Results.

2.2. Similar to 1.2, the authors may want to present the data tracking substrates and metabolites during the xyl-anaerobic test cultures. Especially, near the time point of the sampling for all transcription analyses. And please display error bars on the growth profiles.

We thank the reviewer for this suggestion. We have done similar tracking in our past work (see Myers et al. PLoS Genetics 2019). We opted not to show error bars on the growth curves since they obscure the figure; instead, we annotate the statistical significance that is based on those replicates and their inherent paired, day-to-day variation – it is the statistical significance that is important here and thus for clarify we stick with that presentation.

3. L487: It is difficult to see the clear difference between EWY55 and OPI1 deletion strain regarding growth on solid xylose medium (Fig 5B). Liquid culture, like Fig 5A, would be a better design for comparing their growth patterns.

Thank you for this suggestion – we now show results from liquid growth curves that clearly shows that deletion of *OPI1* from evolved strain EWY55 partially reverts the phenotype back to the unevolved parent: EWY55 *opi1∆* shows statistically significantly reduced anaerobic growth on xylose compared to the EWY55 strain but retains the robust xylose fermentation phenotype. This new work unambiguously shows that Opi1 contributes to the evolved strain phenotype.

4. The authors also may want to present the growth data with cell biomass (dry cell weight) and CFU, which can be more suitable than OD at 600nm to argue the core messages in this study with better resolution considering the outcome of the mutants.

Dry cell weight is a useful measure that we have used in the past (see Wagner et al. PLoS ONE 2019). In our strain and under these conditions, it is highly correlated with optical density. Therefore, we have opted to use OD for this work. Past results have indicated that even though *bcy1-* cultures are not increasing in density, the cells retain viability as indicated by CFU.

Minor issue

1. Introduction: It would be great if the authors specify references per each information (sentence) rather than chunky sentences with multitudinous references.

We attempted to streamline some of the references for readability.

2. L201: “, respectively” seems redundant.

We thank the reviewer for pointing this out; the text has been fixed.

~~~~~~~~~~~~~~

Reviewer #3: In this manuscript, the authors investigate an interesting observation that they had made in a previous work that different mechanisms of activating PKA (either deletion of IRA2, or deletion of BCY1) effect the ability of an engineered strain to ferment xylose and grow. Specifically the IRA2 deletion increases cAMP levels which then causes activation of PKA. This allows the engineered strain to ferment xylose and grow robustly. In contrast, deletion of BCY1, the regulatory subunit of PKA which allows the active subunits of PKA to become constitutively active regardless of cAMP levels, allows some fermentation of xylose, but has very slow growth.

This is a very interesting and important phenotype which these authors have published before and understanding how nuances in the regulation of PKA can allow metabolism and growth to be decoupled is of broad general interest. The fact that they do it in a model system (anaerobic xylose fermentation) which may have useful applications in bioproduction from renewable plant matter is also appealing. The authors produce an important and high-quality transcriptomic dataset, and a lower quality lipidomic dataset and analyse these data to make progress in understanding this mystery. Although their hypothesis that a bottleneck in lipid production is preventing growth in the BCY1 deletion strains is intriguing, I am not fully convinced by the evidence for this. They complement this systems-phenotypic approach with an investigation of strains that have overcome slow growth during laboratory evolution. Although they identify a few potential causative mutations, and find evidence that one mutation is relevant to their phenotype, I am not convinced that this mutation is they key reason that the adapted strain has regained growth, and given that the other strains they recovered have different mutations, there may be many other potential mutations that would allow the strain to retain growth.
Despite these limitations, I believe this study is worthy of publication provided that the authors moderate some of their claims and ensure that the text accurately reflects the data they present. I also found several places where the manuscript was difficult to read and confusing because of poor and possibly inaccurate word choice.

We thank this reviewer for their excitement about the work and their positive assessment.

The authors first present transcriptomic data comparing anaerobic growth of strains with deletions in IRA2 and BCY1 or both genes with either xylose or glucose as a carbon source. They first compare expression in the IRA2 delete (the one strain that could grow) vs. the other three (which could not) and only find two genes differentially expressed in that strain vs the other three analysed as a group. They find that the gene expression of the IRA2 deletion strain is more similar to the non-fermenting parental strain than the fermenting BCY1 deletion strain. They do see that IRA2 deletion results in weakly repressed cyclin CLN2 and CDC20 expression while those genes are more strongly expressed in the other strains, but they suggest that these differences are not sufficient to explain the observed differences in growth.

Strikingly, the authors observe “no correlation of RP and RIBI transcript abundance or response with growth phenotypes”, as the BCY1 delete and IRA2/BCY1 double delete show very little decrease in RP and Ribi transcripts in response to xylose, while the IRA2 delete, which is growing, has a strong decrease.

The authors then compare expression in the non-fermenting strain to the fermenting strains and find many more genes that are differentially expressed. They find that several key metabolic genes are induced strongly in the parental strain but not in the deletion mutants consistent with the hypothesis that ‘the three xylose fermenters recognize xylose as a fermentable carbon, whereas Y184 [the parental strain] does not’.

The next comparison was between the BCY1 deletion strain and the IRA2 deletion strain to see how growth and metabolism were decoupled. In this comparison, the authors looked for enrichment of various functional terms, targets of transcriptional regulators and presence of predicted Transcription Factor motifs in a gene’s promoter. This analysis implicated various regulators with more or less well studied links to PKA including INO4 which is involved with phospholipid biosynthesis. This observation and the fact that many lipid biosynthesis genes were present in genes differentially expressed between BCY1 and IRA2 deletion strains led the authors to conduct lipidomics assays on the cells.

The lipidomics assays were not very reproducible between replicates, but the authors argued that there was a trend in which PC lipids were lower after the xylose shift in BCY1 deletion cells compared to IRA2 deletion cells. They argued that this combined with transcriptomic evidence and phosphorylation status indicated a bottleneck in the PC lipid biosynthesis pathway in BCY1 delete cells. They attempted to rescue this bottleneck with phospholipid precursors, but this only had a minor effect on growth of BCY1 delete cells in xylose.

The authors then conducted laboratory evolution in BCY1 Deletion strains to see if they could identify mutations that rescue growth in xylose. They found several colonies with differing point mutations, copy number variations and aneuploidies that grew better in xylose including one (EWY22) that grew even better than the IRA2 deletion strain. They found that deleting one of the genes altered in that strain caused the evolved strain to grow slightly more slowly.

They then gathered transcriptomic and lipidomic data in that evolved strain and surprisingly the transcriptome and lipidome of that strain was very different from that of the IRA2 delete strain that could also grow in xylose. They argued that the lipidome of the EWY strain overcame the hypothesized bottleneck in PC production in some way distinct from IRA2 delete cells.

The discussion has a very nice section describing how PKA induction may be different between the IRA2 deletion strain and the BCY1 deletion strain. I find myself wishing that the authors had pursued some of these hypotheses more with their experiments to get at why those two perturbations that presumably act in the same direction on the same pathway contribute to the phenotypic differences they observe in the different strains.

I believe the following points are critical problems that ought to be addressed before publishing:

1. The authors state that the IRA2/BCY1 double deletion mutant behaves phenotypically like the BCY1 double deletion mutant, but Fig S1B shows that it actually does not ferment xylose unlike both single deletion strains (Line 142). In fact, the authors state that the double deletion strain is fermenting. Is there an error in FIG S1B? If not, what is the evidence that that strain ferments xylose? I believe that this difference was not pointed out anywhere else in the text. I wrote the rest of this review as though the IRA2/BCY1 is fermenting xylose, as that is an assumption underpinning much of the interpretation of the paper, but I am not convinced that that assumption is true, at least based on the presented evidence.

We apologize that the wrong supplemental figure was included in the original draft. As part of our revisions, we recollected fermentation data for the double-deletion strain and this information is now included in the main Figure 1. We found that the double mutant has a very reproducible growth inhibition when shifted to xylose anaerobically; for reasons that are not clear, the xylose fermentation phenotype was variable day to day: one some days the strain ferments just as the *bcy1-* strain, but on other days fermentation is inhibited. This may be due to extremely high levels of PKA activity, which can be inhibitory for fermentation of other carbon sources. Nonetheless, since our paper focuses on the growth decoupling of the *bcy1-* strain, and since the double mutant was largely used as a genetic approach to compare to the single-gene deletions, the variability of the xylose fermentation aspect of the phenotype does not affect any of our conclusions. To be safe, we redid one analysis relating gene expression to xylose fermentation by comparing the two robustly xylose-fermenting strains (*ira2-* and *bcy1-)* to the non-fermenting Y184 strain; although the number of genes is different due to differences in statistical power, all of the trends and conclusions are the same. All of the other conclusions from our analysis stand, and the analysis of evolved strain EWY55 and EWY55 *opi1-* corroborate many of the hypotheses made based on the *bcy1-* strain.

2. The authors interpret their lipidomic and transcriptomic analysis (e.g. in Line 388) as generating a bottleneck in BCY1 deletion strains in the pathway leading to PC synthesis. However PDME lipids immediately upstream of PC are shown to increase actually increase in the BCY1 deletion mutants. Lower expression of OPI3 in that pathway might support their interpretation, but if so the authors should explain why that gene’s repression can lead to an increase in one of its products (PDME) and a decrease in the other (PC). I admit I am not well versed in lipid biosynthesis, but the omics evidence, combined with the fact that only a very minor increase in growth was achieved when lipids were added back to relieve the bottleneck makes it hard to believe that interpretation. Also when the authors measure the lipidome in the evolved strain which can achieve growth, they suggest that the bottleneck has been overcome (because they have increased levels of DG an TG storage lipids), however there are still decreased amounts of some of the key lipids that they implicate in the bottleneck. The authors suggest that the bottleneck is therefore overcome in some unknown way, but perhaps this evidence should cause them to question the existence or nature of the bottleneck in the first place.

The figure presents a simplified view of the phospholipid pathway, and there are multiple routes to producing and consuming PC beyond Opi3 activity. Thus, a simple explanation is that PDME precursor(s) accumulate while PC is depleted through other pathways, such as the GPC pathway that recycles PC. Supplementing inositol and choline did not reverse the growth arrest; while this supplementation may increase PC production, it would not alleviate the accumulation of PDME which is known to be toxic to cells. Thus, we proposed in the Discussion the possibility of a bottleneck leading to accumulation of PDME to arrest growth. We agree that this is one of several possibilities, and therefore we clarified the text to de-emphasize the bottleneck model and state that flux analysis will be required to prove that possibility.

In general for the lipidomic measurements (which I admit I am not very familiar with), it is disconcerting that the authors are making conclusions based on very few examples that are confidently classified (in Fig 4A, of the 18 lipids that changed, only two seem to have been confidently classified, and it seems to be a similar case for the other lipidomic analysis). It seems unwise to draw conclusions based on these few confidently classified lipids when the majority of changes are from lipids that are not confidently classified.

It is true that a known limitation of lipidomics is that for some lipid classes it is difficult to identify the precise position of unsaturated bonds, leading to their classification into families instead of discrete molecules. The “unknown” lipids present in this dataset were further analyzed with LIPID MAPS, classifying the unknown lipid in the PS, PE, PC family but not able to distinguish between these lipid classes. For this reason, we focus on those lipids that can be clearly identified and placed into the pathway shown in Fig 4C and note in the discussion that more studies on the phospholipid biosynthesis pathway are needed. We highlight that inferences made from the lipidomics along with gene expression analysis from both the *bcy1-* and evolved EWY55 strain repeatedly implicate differences in lipid metabolism across the strains; the new results that clearly show that deletion of *OPI1* from EWY55 partially reverts the phenotype to the *bcy1-* parent are also consistent with these implications.

Finally, these lipidomic measurements seem less reproducible (i.e. than the transcriptomic measurements). The authors helpfully point this out in line 369 mentioning that one of their three BCY1 deletion replicates was not similar to the other two. In that case the authors ought to show the data from all the replicates as they did for the transcriptomic data in Figs 2 and 3. They did show the replicates in Fig S4, but in the text they said that the lower abundance of PC lipids was ‘reproducible’ (lines 386-388) which was not very apparent to me in the figure. In one of the replicates of BCY1 the PC lipids seem to have increased according to that figure.

The reviewer is correct that lipidomic analysis is much more challenging than transcriptomic analysis, and thus we relieved carefully on statistical analysis of the data. In response to the reviewer request, we updated Figure 4 to show individual lipidomics replicates; we highlight that lipids discussed in the text were analyzed statistically considering *bcy1* and *ira2bcy1* strains together, and thus the conclusions in the paper are statistically robust.

A minor related point on this topic: as the comparison described in the text is between the IRA2 delete and the BCY1/IRA2 double delete, the key for Fig 4C should indicate that lipids colored in green were higher in the double delete rather than higher in the single delete (similar for the pink label).

Thank you for the point, we have corrected this in the figure legend to indicate that the analysis was done comparing the *ira2∆* strain and the *ira2∆bcy1∆* strain.

3. I am not convinced that the point mutation the authors saw in the OPI1 gene in strain EWY55 is causal for the growth phenotype. First, it would be helpful if growth of the OPI1 deletion strain were shown over time (as in Fig 5A) so one could evaluate how much of an effect that deletion has on the phenotype. Secondly, the authors point out that there is a chromosome 1 duplication. It seems that this may have an important effect on the phenotype. The authors should at least acknowledge in the text that the chromosome duplication may be important for the phenotype. Could the inability to conduct complementation experiments because introduction of the empty vector could complement low anaerobic growth on xylose on a plate under drug selection be related to this? Perhaps the drug treatment or transformation protocol could give rise to chromosome instability which could lead to restored growth in xylose.

As stated in the text, we were unfortunately unable to confirm phenotypic effects through allele swaps, but we highlighted that it is intriguing that this gene and the pathway it functions in was implicated in multiple analyses throughout the paper. We did take this reviewer’s advice and now provide liquid growth curves along with hplc analysis to quantify xylose consumption over time – the new data clearly show that deletion of *OPI1* from the evolved EWY55 strain partially reverts the phenotype to the *bcy1-* parent: EWY55 *opi1-* shows a significant growth reduction but retains robust xylose fermentation, confirming that Opi1 plays a role.

Aneuploidy was not shared by all strains, and the strains that were aneuploid amplified different chromosomes; thus, a specific chromosome duplication is not contributing universally to the phenotype. It is true that these strains behave differently when grown on plates and in liquid medium, and we considered that the empty vector may be perturbing the phenotypes but this was not supported in various tests.

4. One of the more striking findings of this paper is that the Ribosomal Protein genes and Ribosome Biogenesis genes are not correlated with growth in the various mutants. This is shown in Fig S1B. It seems possible that some of these genes might have altered basal expression in the deletion strains. That information would be good to show in fig S1B (similar to what is shown in fig 2C). In fact, one can see that there is a subset of genes related to translation that have reduced basal expression in the deletion strain. Also, there seems to be a group of translation related genes that have reduced expression in the two strains with BCY1 deletions relative to the IRA1 deletion strain. I would want to see these basal changes before fully believing the claim that the BCY1 deletion strains are unlikely limited by the abundance of RP and RiBi transcripts. Perhaps volcano plots in the supplemental figures showing that there is little change in basal expression in glucose between the different strains for all genes (or at least for these translation related genes) would help to allay this concern.

We have added the basal expression of ribosomal protein/biogenesis genes to Figure S1B, which confirms the data shown in Fig 2C that the basal expression of these genes is not substantially different in the *bcy1-* strains – rather, the *bcy1*- and *ira2bcy1-* strains do not repress the genes’ expression when exposed anaerobically to xylose.

5. In line 220 the authors assert that “there was not a clear gene expression pattern to describe why IRA2 delete cells grow and BCY1 delete cells do not”. I think that the authors should provide a better rationale (perhaps from literature) to explain why the difference they see in cell cycle regulator expression (e.g. CLN2 and CDC20) is not sufficient to explain the difference between the IRA2 and BCY1 deletion strains growth phenotypes. They may be just a few genes, but it is possible that they are important genes. According to SGD, CDC20 is an essential gene and conditional mutants cause cell cycle arrest in M-phase. The cleanest proof would be an experiment in which expression of these regulators is inducibly decreased, or the protein is inducibly degraded in the strains of interest. This would be a difficult experiment, so perhaps the authors would prefer to qualify their assertion to acknowledge that it is possible that these regulators could be the causal difference in expression that they were looking for, but that they don’t have evidence either way.

We appreciate the reviewer’s point and to this end we updated the text to state that these transcripts are well known to fluctuate in abundance with the cell cycle; although these genes couple play a role, their expression differences likely reflect the expected difference between cycling and arrested cells. We updated the text around this point (lines 216-222).

6. One very intriguing hypothesis in the manuscript is that the xylose fermenting strains do not sense xylose as carbon starvation. This hypothesis could stand to be further explored within the data, literature or even with further experiments. One small thing that would be to display the data from Table S6 as a barplot, or even better over a map of the metabolic pathways covered (similar to fig 4C).

This point was a major focus of our past work (Myers et al. PLoS Genetics 2018) and extensively reviewed there; therefore, we did not put a large focus on that here to maintain focus on the current topic of decoupling in the bcy1 strain. Nonetheless, we added Fig 2D and a short discussion to the main text that shows several glucose-repressed genes: these are de-repressed in the Y184 strain, consistent with a glucose starvation response, but not (or to a much lower degree) in the *ira2-* and *bcy1-* strains.

A minor related point is that table S6 (and possibly other tables) rely on a missing external link for the gene name.

Thank you for the suggestion, we have corrected the link.

The following points are meant to help improve the manuscript, but should not hold up publication.

M1. In general the text could use more careful editing for grammar and sentence construction. In several places it appears that the text does not accurately describe what it intends to which makes it hard to follow.
Examples:

a. Line 197-199: We specifically investigated the set of 65 genes encoding cell-cycle regulators… I understood that the 65 genes were not just regulators but were any differentially expressed gene. I think the authors meant to say ‘investigated the set of 65 genes to identify if any encoded cell-cycle regualtors..’ or something like that.

We corrected the text to state, “We specifically investigated the set of 65 genes *for those* *that encode* cell-cycle regulators and kinases …”

b. I don’t understand what the statement in line 153 ‘differences in xylose response drive the differences in fold change response’ it seems circular.

We changed the text to read, “There were major differences in expression comparing the strains growing on xylose, whereas only mild expression differences were observed comparing strains grown on glucose (see Fig 2C, right panel). Correspondingly, strains do not differ substantially in their ability to grow anaerobically on glucose (S1 Fig) (35). Thus, the differences in the fold-change expression response to the carbon shift are driven by differences in the xylose condition. “

c. There is a statement in line 452/453 that contradicts what is shown in the plot. Supplement. The authors state that strain EWY89-1 did not differ significantly from the BCY1 deletion strain, but the plot suggests that it did. Also in fig S6E is BCY1 del really not significantly different from EWY 89-3? This looks like a mistake.

We thank the reviewer for noticing this. We corrected an error in the statistical analysis. EWY89-1 is not statistically different from the *bcy1∆* strain by an ANOVA analysis (p = 0.4854857). EWY89-3 is statistically different from *bcy1∆* by an ANOVA analysis (p = 0.0375332). Both the text and figure have been updated with these corrections.

d. Lines 512-515. Hard to follow and unclear if it was referring to the clusters A and D in fig 5C or not. The number of genes analysed in each barplot for fig 5C would be helpful to show.

We change Fig 5C to a heatmap to better show the gene clusters and added to the figure legend the number of genes in each cluster.

M2. Much of the data described is displayed in heatmaps of various groups of genes clustered in different ways. It is often hard to keep track of how each subset of genes is being chosen. A text label or visual guide in the figures might make it easier to more quickly understand these figures. Also for specific statements in the text volcano plots, scatter plots or bar plots might better illustrate the points. Heatmaps are great for summarizing data, but summaries depicting variability and statistical significance would help better assess specific claims.

A few examples:

a. In line 182, the authors state that only two genes showed xylose responsive expression changes that were specific to IRA2 delete cells. Could those be highlighted or specifically analyzed?

Those genes are mentioned by name (daughter-cell-specific glucanase *DSE4* and L-homoserine-O-acetyltransferase *MET2*) and were identified by robust statistical analysis. Given that their role is difficult to interpret and that we do not pursue them in the paper, we direct the reader to the quantitative values provided in the supplemental tables.

b. Line 232 states that ‘One group of 18 genes (Fig 2C cluster II) is the only cluster in which all three xylose-metabolizing mutants showed one pattern, gene repression, whereas Y184 induced gene expression”. If there is gene repression in the IRA2del mutant in this cluster it is very faint compared to that of the other two fermenting strains. I would need to see something more quantitative (e.g. barplot) to believe this statement.

Again, we respectfully point out that these results are all based on robust statistical analysis. While this group of genes is not a major point of the paper, that there is statistically significant enrichment of protein folding chaperones is interesting and potentially useful for some readers.

c. Line 404 states that Fig 3A shows that BCY1 delete and IRA2/BCY1 delete cells uniquely induce PA biosynthesis genes.

We added a histogram to Fig 3 showing the relative expression of two PA biosynthesis genes in this gene group, *SLC2* and *SCT1*, comparing the log_2_ abundance in YPX versus YPD. As the text states, these genes are uniquely induced in the *bcy1-* and *ira2bcy1-* strains but repressed in *ira2-* cells.

d. The statement in 519-520 describing transcript abundance in EW55 cells vs deletion mutants (possibly a barplot would help).

Due to the number of phospholipid biosynthesis genes, it is not feasible to show the genes as a bar plot, so we direct the reader to S7 Fig which visualizes the comparison of transcript abundance in EWY55 cells compared to *bcy1∆* cells and S16 Table containing quantitative data.

e. In fig 5D it is not clear that the DG is higher in the EW55 strain (line 525). Also the greater induction of TG lipids is not clear – in a few cases it looks just as high as in the BCY1 deletion. It is unclear how the various values for TG from the lipidomics experiments are combined to conclude that TG is greater EWY55 as shown in fig S7 and stated in the text.

We updated Figure 5D to show bar plots of some of the key lipids discussed in the text. We hope this change helps the reviewer. We note that all of the quantitative values are provided in

Table S17.

f. In the conclusion targets of MGA2 are described as being altered in BCY1 delete compared to the

It is unclear what this comment is referring to.

g. In the conclusion, line 582, the fact that INO2/4 targets are repressed is part of the argument for a bottleneck in phospholipid biosynthesis. A separate plot for just these targets might help make this more convincing.

Ino4 targets (which overlap with Ino2 targets) are enriched among the genes that are repressed in *bcy1-* cells but induced in *ira2*; interestingly, there is a smaller set of Ino2/4 targets that are actually repressed in *ira2-* but induced in *bcy1-.* We now provide a new figure (Fig S3C) that shows the expression of the Ino2/4 genes in this group. We added a statement to the text that these differences could reflect complex regulation of a subset of these genes by other regulators; but the result is consistent with our observation that the *bcy1-* strain has unusual regulation of known Ino2/4 genes that suggests atypical regulation.

M3. Some of the methods for analysing the data could use more detailed descriptions (e.g. in the methods section). E.g. what does a comparison to a set of values 'as a group’ mean – are they averaged as though they were replicates before or is this done through a contrast in the edgeR package?

We used specific growth phenotypes (e.g. ability to grow anaerobically on xylose) as factors in the linear model. We clarified the text to state that strains were analyzed as a group in the statistical model. These are all accepted methods in genomics, no data were averaged.

M4. It is hard to interpret the regulatory relationships shown in fig 3B in light of the fact that PKA should be increased in both IRA2 deletion mutants and BCY1 deletion mutants, but the expression of these targets downstream of PKA under a shift to xylose are moving in opposite directions. It would be more helpful to get a picture of how the authors propose that the different ways of increasing PKA activity cause opposing signals to be propagated to these known or suspected PKA targets. Some nice ideas are mentioned in the following paragraph relating to lipid biosynthesis.

We thank the reviewer for this suggestion. We outlined several possibilities in the Discussion; our favored model is that Bcy1 acts as an AKAP in yeast to direct PKA to specific targets, and in the absence of Bcy1 PKA is upregulated but also mis-directed toward some targets and likely away from others. We avoided adding a complicated model figure for the paper, in part because the possibilities are numerous and will require subsequent experiments to elucidate.

Given the lack of enrichment of MGA2 targets (Line 300), it seems unwise to show the link in Fig 3B without stronger evidence. The caption for that figure states that it is showing ‘transcription factors whose targets or known binding sites were enriched in A’. The targets for MGA2 were not enriched, were the known binding sites enriched? If the presence of the canonical target OLE1 is sufficient, then why do a majority of the other targets not get induced? More importantly can the authors show that Mga2 is more active in xylose shift in IRA2 delete cells but less active in a BCY1 deletion strain. This claim is repeated in line 561 referring to fig 3A in the conclusion.

We have removed the Mga2 connection from the main text/Figure 3 and instead added it to the Discussion as another potentially connected transcription factor in the network based on previous work (Myers et al. PLoS Genetics 2019); nearly half of known Mga2 targets were identified in our analysis, even though the statistical significance of the enrichment missed our cutoff.

M5. It would be useful to include enrichment calculations for Table S8 (e.g. how many total genes are in the dataset related to the terms, how many are in the subset of genes analysed, and enrichment calculations such as a hypergeometric test statistic).

Table S8 has been updated with numbers requested by the reviewer.

M6. It is a bit hard to interpret the transcriptional data in figure 4C. Much of this could be because the bold boxes represent significant differences with respect to IRA2 deletion, but not significance in terms of fold change for that sample.

The main point of this section is identifying differences between the *bcy1∆* strains and *ira2∆* strain, which is why we opted to represent bold boxes as significant differences with respect to the *ira2∆* strain. Additionally, it is challenging to find a graphic that combine transcriptomic, lipidomic, and phosphoproteomic data as we have done here. After testing several graphics, this is the clearest representation we landed on. As this type of plot with highlighted boxes is a common approach, we opted for this as the best presentation.

The following statements that rely on 4C were hard to evaluate:
a. Line 336, ‘the gene encoding the PS synthase CHO1 was strongly induced in Y184 cells'

Our point here is that the difference in PS abundance in Y184 is not due to altered expression of the PS synthase *CHO1*, since that genes is highly induced in Y184.

b. Line 396 that the cardiolipin biosynthetic genes were more lowly induced in ira2 deletion cells but repressed or induced in BCY1 deletion cells, especially CLD1.

Our point here is that the differences in cardiolipin levels are mirrored by differences in the cardiolipin metabolism genes.

M7. In table 2 a column showing the growth rate of the strain would be helpful. Also, it is not clear from the text or caption whether this is all the SNPs and rearrangements that were found, or just a selection of potentially interesting ones.

We clarified the Methods / Results to state that only SNPs occurring in the coding region of a gene were considered for further analysis.

M9. PS is listed in the text in line 526 but not shown in Fig 5D.

As referenced in the paper, those data are shown collectively in “S17 Table”
