# Supplementary material for: PKA regulatory subunit Bcy1 couples growth, lipid metabolism, and fermentation during anaerobic xylose growth in Saccharomyces cerevisiae
Source: PLoS Genet. 2023 Jul 6;19(7):e1010593. doi: 10.1371/journal.pgen.1010593 (PMC10353814; doi:10.1371/journal.pgen.1010593)
Supplement: Attachment — Submitted filename: Wagner_PLoSGenetics_ReReviewerComments_final.docx [file pgen.1010593.s025.docx]

Response to Re-Review of Wagner et al.

Editor and reviewer comments are in black text, author responses are in blue text.

Editor Comments:
Two of the reviewers have assessed the manuscript again and appreciate the new data, analyses and other amendments to the work. Overall they are positive. However, they also brought up additional issues and clarifications. Rev 3 in addition to minor points, brought up the variable xylose metabolism of the double mutant with respect to the lipid data, and some questions about the new experiment in fig 5B. While I don’t think collecting additional data would obviously make things more clear, I also think it is reasonable to acknowledge the issues raised in order to give the reader some idea of potential limitations. For example, the double mutant variability could mean certain lipid differences were missed (not significant). Rev 1 also made some suggestions for clarify, but these are certainly discretionary.

**Comments to the Authors:
Please note here if the review is uploaded as an attachment.**

Reviewer #1: Why not include the alternative GEF Sdc25 in Figure 1A?

We have updated Figure 1A to include Sdc25.

The p-values are clear but why are there not error bars on the graphs for Figs 1B and 1C and other growth curve data?

As outlined before, the error bars confound the figure. What is important is the statistical analysis which is more important than deviation bars.

Compared to the very nice heat maps, Figure 4C remains difficult to interpret. There are multiple squares. Some are outlined, others are not. Can the figure be clarified for the reader?

We have done our best to clarify this figure, which shows complex data for four different strain comparisons. The boxes are described in the key and the box outlines are described in the figure legend.

Reviewer #3: I have reviewed the authors response and appreciate the additional experiments and clarifications they have done in the manuscript in response to my comments and those of the other reviewers. I feel like the manuscript has improved and remains of general interest to the field.

I am, however worried that the lipidomics analysis (Figure 4) relies too much on a strain (ira2/bcy1 double mutant) that has a variable xylose metabolism phenotype. Also the new data in fig 5B shows the EWY55 strain growing to much higher densities than any other strains in the paper which is worrying.

I believe both these points ought to be addressed prior to publication.
After rereading the manuscript and their responses, I have the following remaining comments:

Major points:

1) I appreciate that the authors have attempted to verify whether the ira2/bcy1 double mutant ferments xylose. However it is very unsatisfying that that mutant has such a variable xylose metabolism phenotype:

“These data were recollected as part of our revisions: the new data show that
the ira2bcy1 double mutant has a variable xylose metabolism phenotype (on some days
consuming xylose anaerobically as the bcy1- strain and on other days not); but there is an
invariant growth arrest under these conditions.”

The fact that the phenotype is not reproducible makes me worried that there is either a problem with the strain (genome instability or if it picked up additional mutations after first being created) or else with the growth protocol. It is commendable that they redid their analysis of the transcriptomic data leaving that strain out, and I am glad that it did not affect their conclusions. Unfortunately, as they had variable data in their bcy1 deletion mutant, they still rely heavily on data from the ira2bcy1 double mutant for their lipidomics analysis. The problem is that we don’t know what state this mutant was in for the data collected on that experiment. Was it fermenting xylose or not on that day? This seems like it would effect the metabolism significantly, which I assume would effect the lipidome.

The authors also rely on the double mutant to understand gene expression patterns correlated with growth phenotypes (section starting on line 193) which is less problematic because the double mutant does show a strong growth phenotype despite the variable fermentation phenotype.

It seems like there are a few options:
- Get to the bottom of the variably xylose metabolism phenotype and be clear about what state (xylose fermenting or not) the lipidomics data was collected in.
- Conduct the lipidomics analysis using only the two replicates of the bcy1 deletion mutant. This may make it harder to get statistically significant conclusions, but it seems better than relying on data from a strain with an ambiguous and highly variable xylose fermentation phenotype.
- Ideal, but may not be possible for this paper: Repeat the lipidomics experiment with the bcy1 deletion mutant to get enough replicates upon which to base the conclusions and leave out the data from the double deletion strain, or highly caveat any analysis based on that strain.

We appreciate the points made by this reviewer; however, we disagree that the variability in the double mutant strain precludes us from analyzing and interpreting *bcy1Δ* data from the paper. First, both the transcriptomic data and the lipidomic data are highly similar for the *bcy1Δ* and *ira2Δbcy1Δ* strains across multiple replicates; the goal of the paper was to understand the *bcy1Δ* strain’s decoupling, which is very reproducible and robust. To be extra careful about our conclusions, we redid the transcriptomic analysis in Figure 3 omitting the *ira2Δbcy1Δ* data – we got essentially the same results, validating our approach and analysis. Second, it is not uncommon for signaling mutants to have effects on variability and heterogeneity, and in fact our lab has studied this extensively in other recent work. Our lab has very deep experience with these types of experiments, and also with unstable genotypes; we believe it is likely the hyper-active PKA signaling, and not unstable genotypes, that gives rise to this heterogeneity. Finally, we retained the focus on the double mutant for our lipidomic statistical analysis, since the replicates are in fact highly reproducible just as the growth defect is highly reproducible in this strain, and since the response matches the two good replicates of the *bcy1Δ* strain, just as the strain’s transcriptomic response matches the *bcy1Δ* strain. Together, this is consistent with reproducible hyper-activation of the PKA pathway, even though the metabolism phenotype was variable.

Nonetheless, we agree with the editor that a discussion of the caveats of the double mutant strain is warranted. Therefore, we added these statements:

Line 394, “One caveat of this analysis is that the *ira2∆bcy1∆* strain displays a variable anaerobic-xylose fermentation profile; nonetheless, given the similarity to *bcy1∆* phenotypes, the high reproducibility of the double mutant’s transcriptomic and lipidomic profiles suggests a good representation of hyper-active PKA signaling. It is possible that this analysis may miss some lipidomic changes related to the variation in *ira2∆bcy1∆* metabolism profiles.”

In a related point, Figure 4 only shows two replicates of the bcy1deletion data. It is not clear to me whether the data from one of the replicates (the outlier) was thrown out in the analysis altogether (i.e. it was determined that something went wrong with the protocol) or if it was included in the analysis but not shown on the figure. I personally think if it was included in the analysis, it would be best to include it in the figure.

We removed the *bcy1∆* strain’s rep B from the lipidomics analysis, and thus removed that replicate from the figure.

2) While the new experiment in fig 5B is consistent with the author’s interpretation that the OPI1 point mutation is an adaptive change that the EW55 strain acquired to resume growth in xylose despite the bcy1 mutation, I have several worries about that data and experiment:

A. EW55 grows much better in figure 3B than in Figure 3A, though they are ostensibly the same growth conditions. Indeed the scale of the OD for figure 3B seems to be higher than for any other instance of anaerobic growth in xylose. Could there be an undetected difference in the growth conditions? In particular the final OD of the EWY55, OPI1 delete strain is actually higher than the final OD of EW55 in figure 3A.

The new experiment added in Figure 5B was done over a year later than the experiment shown in Figure 5A; it is also from a longer time course. Although the final OD is higher for EWY55 in newer data compared to EWY55 in the older data, this is likely due to new lots of media reagents. The relative trends across *ira2Δ,* *bcy1Δ,* and EWY55 strains are highly consistent in all of our experiments. The important point in this experiment is that deleting *OPI1* from EWY55 reduces growth rate when grown side-by-side with EWY55 in the new experiment, which is the proper control for growth experiments. Therefore, the results clearly show that *OPI1* deletion reduces growth rate compared to the parental strain grown under identical conditions.

B. Could it be that in the process of deleting the OPI1 gene in the EW55 strain, that strain also lost all or part of its duplicated chromosome 1? In that case the effect could be to either the OPI1 gene, the loss of the duplicated chromosome, or a combination of the two factors.

Given all the other indications pointing to Opi1, and since the chromosome amplifications are not shared across strains, we do not believe that the chromosomal aneuploidies have anything to do with this phenotype. We have added a statement to the Results section that we cannot be sure that Opi1 is the causal gene, but that results are consistent with this hypothesis.

Minor points:
Besides those two comments I have a few more minor issues with the remaining text:

1) The authors added figure S1 to make the point in line 150 that: The three strains grow indistinguishably on glucose (p> 0.05, S1A Fig) with similar glucose consumption (p > 0.05, S1B Fig) and ethanol production (p > 0.05, S1C Fig).

I am not exactly sure how the ANOVA was implemented for these growth curves, but to the eye it looks as though the IRA2 delete cell grows slightly worse in glucose, and has lower glucose consumption and ethanol production toward the latter half of the growth curve. In general the growth curves (Fig S1A seem very variable, especially given that these are the mean values of three replicates). If the ANOVA test was done for all the timepoints on the growth curve, I wonder if there might be a bias towards the earlier part of the curve when the differences were much smaller. I have a feeling that at 8 hours and with more replicates, there would be a statistical difference between these strains (at least for IRA2delete) for these phenotypes.

We can assure this reviewer that this experiment was done correctly, we have always seen that these strains grow indistinguishably on glucose and the important point here is that there are no statistical differences across the replicates.

As the gene expression data was collected after the cells were grown on glucose for only 5 hours, when the differences do look small, I presume that this difference will not alter their conclusions. Perhaps qualifying the ‘indistinguishable’ statement with ‘indistinguishable between 4 and 6 hours’ would be more accurate.

The statistics apply to the entire time course so we prefer to leave the language as is in the paper, which is that there is no statistical difference between the strains’ growth patterns.

2) The new text in Line 161 says:

“There were major differences in expression comparing the strains growing on xylose,
whereas only mild expression differences were observed comparing strains grown on glucose (see Fig 2C, right panel).”

Referring to Figure 2C as evidence for this statement is somewhat circular as the 292 genes chosen for that figure were selected because they had differences in expression in the glucose to xylose shift between Y184 and the other two robustly fermenting strains. I imagine that would enrich for genes that change a lot under xylose between the strains. More convincing for this point would be to look at expression differences between strains grown in glucose for all genes (e.g. differences in basal expression values between strains for all the genes with data – thus showing basal data for S2A).

The point of this control is to show that our selection of genes differentially expressed on xylose are due to the xylose condition and not driven by the starting glucose conditions. This figure shows clearly that these genes were selected for their xylose response, which was the main point.


3) In figure S3B two separate motifs are shown. It is not clear from the figure or the caption what those motifs are. It appears from that the short one on the top is the AFT1/2 consensus site taken from the Meme Suite Tomtom program (possibly Yamaguchi-Iwai Y et al., EMBO J, 1996 Jul 1;15(13):3377-84), and I am guessing that the long, lower motif was empirically identified using MEME on the promoters of the genes repressed in the BCY1 delete strain on a shift to Xylose.

We have updated the figure caption to clarify which motif is which.

4) Line 353: I assume the 18 lipids were identified out of the 239 confidently identified lipids rather than the overall total of 4000 – it would be helpful to clarify that in the text.

We updated the text to clarify.

5) Line 361 states:
“Instead, we analyzed previous phosphoproteomic data from our lab and discovered that Cho1 was phosphorylated to a much higher degree in the Y184 strain on serine 46 (|log2FC| > 2, Table 1)”

Perhaps I am reading table 1 incorrectly but it appears that the average log2 fold change for Cho1 S46 in Y184 compared to the two deletion strains was lower than 2: 1.08 (vs ira2del) or 1.43 (vs bcy1del)?

We apologize for the typo, we corrected to clarify that the cutoff was a log2 fold-change of 1, not 2.

6) Line 534: Although many of the same genes were affected, expression in the EWY55 strain was actually more dissimilar to ira2del than the parental bcy1del strain (Fig 5C)

It is not clear to me how Fig 5C shows this. I feel like a comparison between EWY55 and IRA2 delete strain is missing.

We changed the text to clarify that the EWY55 expression does not recapitulate the ira2∆ strain’s expression when compared to the bcy1∆ strain. The text now reads (line 545) “This indicates that the evolved EWY55 did not recouple growth and metabolism under anaerobic xylose conditions via reverting to the *ira2∆* strain’s expression patterns.”

7) In response to one of my minor comments from the review:
Line 232 states that ‘One group of 18 genes (Fig 2C cluster II) is the only cluster in which all
three xylose-metabolizing mutants showed one pattern, gene repression, whereas Y184
induced gene expression”. If there is gene repression in the IRA2del mutant in this cluster it is very faint compared to that of the other two fermenting strains. I would need to see something more quantitative (e.g. barplot) to believe this statement.

Author's response:
“Again, we respectfully point out that these results are all based on robust statistical analysis.
While this group of genes is not a major point of the paper, that there is statistically significant enrichment of protein folding chaperones is interesting and potentially useful for some readers.”

After the authors redid the analysis, cluster II now contains more genes and the statement is on Line 252:
Cluster II contained 31 genes induced in Y184 and repressed in both ira2Δ and bcy1Δ strains. This group was enriched for genes involved in protein folding (p = 9.49x10-6, hypergeometric test)

While it is clear from the heatmap that these genes are repressed in the BCY2 delete mutant, the authors seem to claim that these genes are all repressed in IRA2 delete mutant. That does not appear to be the case. Instead it looks like the cluster shows no change in IRA2, an increase in Y184 and a decrease in BCY2 in general. I could not easily find the genes identified in cluster 2 in the supplemental table so I could not verify this.

We changed the text to clarify. The text now reads, (line 253) “Cluster II contained 31 genes induced in Y184 and either unchanged or repressed in both *ira2∆* and *bcy1∆* strains.”

8) I suggested in my review that the following statement could be better illustrated with a bar graph:

Line 545: “In general, EWY55 cells showed lower transcript abundances of phospholipid
biosynthesis genes compared to the bcy1Δ strain grown anaerobically on xylose (S16 Table), 547 making its expression even more divergent from the ira2Δ strain.”

“Due to the number of phospholipid biosynthesis genes, it is not feasible to show the genes as a bar plot, so we direct the reader to S7 Fig which visualizes the comparison of transcript
abundance in EWY55 cells compared to bcy1Δ cells and S16 Table containing quantitative
data.”

a) I believe that S16 Table does not have transcript abundance data, but rather lipidomics data.

b) I was not suggesting a bar plot for every single phospholipid biosynthesis gene, but rather a summary – one bar for each strain summarizing transcript abundances of all phospholipid biosynthesis genes (ideally normalized first for each gene). Alternatively the authors could state the numerical levels of transcript abundance, or the percentage of phospholipid biosynthesis genes that were higher in EWY55 compared to bcy1delta in the text, and a statistical test to show that they are different. This is a minor point – just something that as a reader I cannot assess easily based on the data shown and just have to take on faith, and the information shown on a small subset of genes highlighted in S7 Fig.

We apologies for the confusion. We have updated S16 Table with the correct values. We appreciate the reviewer’s assessment that this is a minor concern, so we direct the reader to data in S16 Table for the quantitative values.
